# Sex and age-related implications for preventive measures of intensive care admitted traumatic brain injury patients in Switzerland: an observational study

**DOI:** 10.1007/s00423-025-03720-w

**Published:** 2025-05-03

**Authors:** Juliane Fleischer, Giovanna Brandi, Henrik Teuber, Sarah Flückiger, Stefan Y. Bögli, Simone Unseld

**Affiliations:** 1 Institute of Intensive Care Medicine, University Hospital Zurich, Rämistrasse 100, 8091 Zurich, Switzerland; 2 Department of Traumatology, University Hospital Zurich, Zurich, Switzerland; 3 Department of Neurology, Clinical Neuroscience Centre, University Hospital Zurich, Zurich, Switzerland

**Keywords:** Traumatic brain injury, Intensive care medicine, Epidemiology, Sex difference, Prevention

## Abstract

**Purpose:**

Epidemiological studies of traumatic brain injury (TBI) in Switzerland have, to date, poorly investigated sex-related differences in causality and predisposing factors. This study examines differences in sex and age related TBI epidemiology in a high-volume trauma centre intensive care unit (ICU) cohort, aiming to identify potential targets for prevention.

**Methods:**

This retrospective, single centre study includes all consecutive TBI patients admitted to the ICU in a 4-year study period. Patient demographics, comorbidities, co-medication, trauma setting and associated risk behaviour were compared between sexes and age groups (over/under 65 years).

**Results:**

592 patients (73.3% male, 26.7% female) were included. The leading cause of TBI was falls (52.4%), followed by road traffic accidents (RTA) (35.8%). Overall, men were more likely to suffer from a road traffic accident, while women were more likely to suffer a low energy fall. No differences in injury severity and comorbidities between sexes were observed. Young patients most likely suffered from a RTA while older patients from a low energy fall irrespective of sex. Both sexes portrayed risk associated behaviors with higher rates of alcohol intoxication in males, while females were less likely to wear a helmet in two-wheeled RTAs.

**Conclusions:**

We conclude that sex- and age-related epidemiologic differences in TBI exist. Our results suggest that sex and age-specific prevention measures might be advisable for optimal mitigation of TBI and its sequelae.

## Background

Traumatic brain injury (TBI) continues to be a significant cause of morbidity and mortality worldwide, with relevant public health and social impact contributing to societal disease burden and loss of productive years [1–3]. The epidemiology of TBI is well described in the developed world [2, 4–6], with socio-economic, cultural, and geopolitical factors impacting differences in local incidence, trauma mechanisms, and long-term outcomes. Sex, age, and gender-specific factors play a role in the epidemiology of TBI [7]. For example, men are more prone to suffer a work related TBI or a road traffic accident (RTA), while women are at a higher risk of TBI due to violence in interpersonal relationships [8–10]. Furthermore, falls are the most predominant cause of TBI in older patients [11].

Switzerland is a well-developed central European country ranked highest in the global Human development index (HDI) [12] and 13 th in the global gender gap index in 2022 [13]. Few studies describing the epidemiology of TBI in Switzerland exist [14–17]. Sex-related differences in causality have not been investigated in-depth. To date, only one study has described sex-related differences in a Swiss TBI cohort [18]. As growing evidence supports improved outcomes with personalized care[19], we aimed to identify specific potential prevention targets in different patient populations, focusing on epidemiologic causality differences between the sexes.

## Materials and methods

This is a single-center, retrospectively analyzed registry-based observational study conducted at the Institute for Intensive Care, University Hospital of Zurich in Switzerland. All consecutive TBI patients [20] admitted to the ICU between January 2018 and August 2021 were screened for eligibility.

### Inclusion and exclusion criteria

All adult patients (≥ 18 years of age) with TBI admitted to the ICU of the University Hospital Zurich were included. Patients were excluded if the time interval between trauma and hospital admission was greater than 24 h, or if they refused analysis of their data (documented written or oral refusal).

### Clinical management

All patients were treated according to current best practice and an institutional clinical protocol based on the Brain trauma foundation guidelines [21], focusing on the prevention of secondary brain injury. An interdisciplinary team of ICU consultants, neurosurgeons and trauma surgeons were responsible for clinical decision-making.

### Data collection

The registry (built within the Research electronic data capture (REDcap; Vanderbilt University, Nashville, USA)[22, 23]) was established retrospectively and data were collected from the electronic medical record (KISIM-TM; Cistec® Zurich, Switzerland) and the electronic patient data management system (MetaVision Suite; iMDsoft®, Tel Aviv, Israel). Demographic data collected includes age, sex, comorbidities based on the Charlson Comorbidity Index (CCI)[24], prior chronic medication, clinical frailty index [25], living situation (alone, with other persons at home, retirement home, nursing home) and level of social or physical dependence (independent vs. dependency on ambulatory social or medical services).

The injury event was characterized by location and setting (rural, urban, indoor, outdoor, home environment, workplace, school, at sports or leisure activities, military setting) and cause (RTA, fall, criminal activity, and others). In the case of RTAs, a distinction of the involved transport mode was recorded (car, motorcycle, e-scooter, bicycle, e-bicycle or pedestrian) as well as the usage of safety equipment (cars: usage of seatbelts; other modes of transport: usage of helmets). The incidence of concomitant drug use (alcohol and illicit substances), self-inflicted injuries, and attempted suicide was also recorded.

Injury Severity Score (ISS) [26] and Abbreviated Injury Scale (AIS) [27], as well as initial Glasgow Coma Scales score (GCS) [28, 29] were used to assess injury severity. Lastly, in-hospital mortality rate was noted.

### Methods

Statistical analysis was performed using R version 3.3.1. Data was dichotomized by sex (male vs. female). A subgroup analysis was performed describing different age groups (over/under 65 years). Consequently, analysis between sex and between sex and age was carried out. Data are reported as counts/percentages, or as median including the interquartile range (IQR) as appropriate [30]. All continuous data were tested for normality using Shapiro–Wilk's test. Categorical or ordinal variables were compared using Pearson's χ2 or Fisher's exact test, while continuous variables were compared using Student's t-tests or Mann–Whitney U tests for parametric and non-parametric data, respectively, where appropriate [31, 32]. The significance level was set at p < 0.05. Our primary aim was to describe sex related differences relevant for prevention rather than the effect of these differences on outcome. Consequently, we abstained from performing a multivariable analysis.

## Results

592 consecutive patients (73.3% male) with a median age of 56 (IQR 35–74) years were included in this study (Tables 1 and 2). Men were significantly younger, with a median age of 53 vs. 65 years (p = 0.001). Women were less likely to be independent (82.3 vs. 91.4%, p = 0.01). Furthermore, women had a significantly higher incidence of psychiatric comorbidities (21.5% vs 12.1%, p = 0.014) and were taking psychotropic co-medication more frequently than men (26.8% vs 17.1%, p = 0.012). Regarding trauma setting, women were more frequently injured indoors (37.3% vs 22.1%, p < 0.001) and within the home environment (44.9% vs 22.6%, p < 0.001), while men were more frequently injured in a rural setting (26% vs 16.5%, p = 0.02). The most frequent trauma mechanism in men was RTAs (37.9%). Low energy falls were the most common trauma mechanism in women and were significantly more common versus men (44.9 vs. 28.8%, p < 0.001). Men were more frequently under the influence of alcohol at the time of trauma than women (23.3% vs 12%, p = 0.004). Women suffered two-wheeled RTAs while not wearing a helmet significantly more frequently than men (57.1 vs. 22.8%, p = 0.045).

**Table 1:** Whole population: demographics, injury severity and outcome

Whole population (N = 592)		Male (73.3%)	Female (26.7%)	*p*-value
Age		53 (33—71)	65 (49–79)	** < 0.001**
Living situation	alone at home	109 (25.1%)	52 (32.9%)	0.712
	with other persons at home	263 (60.6%)	77 (48.7%)	0.118
Support in everyday life	independent	395 (91.4%)	130 (82.3%)	**0.01**
Comorbidities	psychiatric diagnosis	56 (12.1%)	34 (21.5%)	**0.014**
	alcoholism	86 (19.8%)	29 (18.4%)	0.779
	smoking	74 (17.1%)	29 (18.4%)	0.804
Charlson Comorbidity Index	total	0 (0—1)	0 (0—1)	0.58
Injury Severity Score		22 (14—29)	24.5 (16—30)	0.864
Abbreviated Injury Score	Head and Neck	3 (2—4)	3(3—5)	0.166
	Facial	0 (0—2)	0 (0—1)	0.448
	Chest	0 (0—3)	0 (0—3)	0.523
	Abdomen	0 (0—0)	0 (0—2)	0.679
	Extremity, pelvis	0 (0—2)	0 (0—2)	0.185
	Other	1 (0—1)	1 (0—1)	0.058
First preclinical GCS		13 (7—15)	13 (6—14)	0.171
Outcome				
Alive at discharge		370 (85.3%)	115 (72.8%)	** < 0.001**

^*^Data shown as number (%) or as median (IQR)

**Table 2:** Whole population: Trauma setting, mechanism, risk behaviour and co-medication

Trauma setting		Male (73.3%)	Female (26.7%)	*p*-value
Rural		113 (26.0%)	26 (16.5%)	**0.02**
Urban		103 (23.7%)	34 (21.5%)	0.649
Home environment		98 (22.6%)	71 (44.9%)	** < 0.001**
Workplace/school		29 (6.7%)	2 (1.3%)	**0.006**
Indoor		95 (22.1%)	59 (37.3%)	** < 0.001**
Trauma mechanism				
Road traffic accident	total	164 (37.9%)	47 (29.9%)	0.754
	car	33 (7.6%)	10 (6.3%)	
	motorcyclist	52 (12.0%)	8 (5.1%)	
	e-scooter	6 (1.4%)	0 (0.0%)	
	bicycle	50 (11.5%)	10 (6.3%)	
	e-bike	10 (2.3%)	3 (1.9%)	
	pedestrian	12 (2.8%)	16 (10.1%)	*
Railway accident		17 (3.9%)	0 (0.0%)	
Fall	low energy fall < 2 m	125 (28.8%)	71 (44.9%)	** < 0.001**
	high energy fall > 2 m	90 (20.7%)	31 (19.6%)	0.855
Criminal activity		22 (5.1%)	1 (0.6%)	0.137
Gunshot		7 (1.6%)	1 (0.6%)	0.688
Burial/Avalanche		4 (0.9%)	2 (1.3%)	0.66
Risk behavior and co-medication				
Under influence of alcohol		101 (23.3%)	19 (12.0%)	**0.004**
Under influence of other substances		25 (5.8%)	4 (2.5%)	0.133
Self-inflicted injury		30 (6.9%)	16 (10.1%)	0.263
Not belted		8 (24.2%)	1 (10.0%)	0.623
Not helmeted	total	39 (22.8%)	9 (57.1%)	**0.045**
	motorcyclist	4 (7.7%)	0 (0.0%)	
	e-scooter	6 (100.0%)	0 (0.0%)	
	cyclist	24 (48.0%)	8 (80.0%)	
	e-bike cyclist	5 (55.6%)	1 (33.3%)	
Prior medication	psychotropics	74 (17.1%)	42 (26.8%)	**0.012**
	anticoagulation	35 (8.1%)	21 (13.4%)	0.074
	antiaggregation	76 (17.5%)	23 (14.7%)	0.485

^*^Data shown as number (%); significant results in the subgroup analysis are with an asterix (*)

### Younger population (age < 65 years)

In the younger population (Tables 3 and 4), epidemiologic and trauma-associated factors were similar compared to the whole patient population. Specifically, median age, incidence of psychiatric diagnoses, and rate of psychotropic co-medication was higher in women versus men (49 years vs. 38 years, p = 0.035; 32% vs 14%, p < 0.001; 26.3% vs 13.3%, p = 0.01, respectively). Younger women were also more frequently injured at home (32.2% vs 14.7%, p = 0.001). Relative to the whole population, trauma mechanisms were comparable between sexes with the primary cause of TBI being RTAs in both sexes.

**Table 3:** Younger Population: Demographics, injury severity and outcome

< 65 years (N = 370)		Male (79.9%)	Female (20.1%)	*p*-value
Age		38 (29—54)	49 (33–55)	**0.035**
Living situation	alone at home	83 (28.3%)	22 (28.6%)	1.00
	with other persons at home	161 (54.9%)	41 (53.2%)	1.00
Support in everyday life	independent	279 (95.9%)	72 (93.5%)	0.43
Comorbidities	pyschiatric diagnosis	42 (14%)	25 (32%)	** < 0.001**
	alcoholism	64 (21.8%)	19 (24.7%)	0.706
	smoking	55 (18.8%)	20 (26.0%)	0.215
	ilicit drug use	38 (13.0%)	8 (10.4%)	0.677
Charlson Comorbidity Index		0 (0—0)	0 (0—0)	0.428
Injury Severity Score ISS	total	22 (16—29)	24 (14—29)	0.93
Abbreviated Injury Score	Head and Neck	3 (2—4)	3 (2—4)	0.633
	Facial	0 (0—2)	0 (0—1)	0.64
	Chest	1 (0—3)	(0—3)	0.603
	Abdomen	0 (0—2)	0 (0—2)	0.517
	Extremity, pelvis	1 (0—2)	2 (0—2)	0.07
	Other	1 (0—1)	1 (0—1)	0.113
First preclinical GCS		13 (8—15)	12 (5—14)	0.461
Outcome				
Alive at discharge		268 (91.5%)	63 (81.8%)	**0.025**

^*^Data shown as number (%) or as median (IQR)

**Table 4:** Younger population: Trauma setting, mechanism, risk behaviour, and co-medication

Trauma setting		Male (79.9%)	Female (20.1%)	*p*-value
Rural		88 (30.0%)	17 (22.1%)	0.216
Urban		73 (24.9%)	19 (24.7%)	1
Home environment		43 (14.7%)	24 (31.2%)	**0.001**
Workplace/school		28 (9.6%)	2 (2.6%)	0.058
Indoor		42 (14.5%)	15 (19.5%)	0.368
Trauma mechanism				
Road traffic accident	total	122 (41.8%)	31 (40.8%)	0.28
	car	25 (8.5%)	5 (6.5%)	
	motorcycle	42 (14.3%)	8 (10.4%)	
	e-scooter	5 (1.7%)	0 (0.0%)	
	bicycle	33 (11.3%)	9 (11.7%)	
	e-bike	7 (2.4%)	2 (2.6%)	
	pedestrian	9 (3.1%)	7 (9.1%)	
Railway accident		14 (4.8%)	0 (0.0%)	
Fall	low energy fall < 2 m	55 (18.8%)	19 (24.7%)	0.321
	high energy fall > 2 m	67 (22.9%)	23 (29.9%)	0.26
Delict		21 (7.2%)	1 (1.3%)	0.543
Gunshot		5 (1.7%)	1 (1.3%)	1
Burial/Avalanche		4 (1.4%)	1 (1.3%)	1
Risk behavior and co-medication				
Under influence of alcohol		81 (27.6%)	14 (18.2%)	0.122
Under influence of other substances		23 (7.9%)	4 (5.2%)	0.622
Self-inflicted injury		26 (8.9%)	13 (16.9%)	0.068
Not belted		7 (28.0%)	0 (0.0%)	
Not helmeted	Total	31 (35.6%)	8 (42.1%)	0.281
	motorcyclist	4 (9.5%)	0 (0.0%)	
	e-scooter	5 (100.0%)	0 (0.0%)	
	cyclist	17 (51.5%)	8 (88.9%)	
	e-bike cyclist	5 (71.4%)	0 (0.0%)	
Prior medication	psychotropics	39 (13.3%)	20 (26.3%)	**0.01**
	anticoagulation	9 (3.1%)	2 (2.6%)	1
	antiaggregation	15 (5.1%)	3 (3.9%)	1

^*^Data shown as number (%)

### Older population (age ≥ 65 years)

When looking at the older population cohort (Tables 5 and 6), there were considerable differences in living situation. Older women were living more commonly alone than men (37% vs 18.4%, p = 0.026). They were more frequently injured indoors (54.3% vs 37.9%, *p* = 0.025) and within the home environment (58% vs 39%, *p* = 0.009). Compared to the younger population, there was a considerable shift in cause of trauma with low energy falls being the primary cause of TBI in both sexes.

**Table 5:** Older population: Demographics, injury severity and outcome

> 65 years (*N* = 222)		Male (63.5%)	Female (36.5%)	*p*-value
Age		78 (72—84)	79 (72—85)	0.082
Living situation	alone at home	26 (18.4%)	30 (37.0%)	**0.026**
	with other persons at home	102 (72.3%)	36 (44.4%)	** < 0.001**
	retirement home	8 (5.7%)	7 (8.6%)	1.00
	nursing home	2 (1.4%)	2 (2.5%)	1.00
Support in everyday life	independent	116 (82.3%)	58 (71.6%)	0.379
Estimated Clinical Frailty Index		3 (2—4)	3 (2—4)	0.593
Comorbidities	psychiatric diagnosis	14 (9.9%)	9 (11.1%)	0.961
	alcoholism	22 (15.6%)	10 (12.3%)	0.641
	smoking	19 (13.5%)	9 (11.1%)	0.764
Charlson Comorbidity Index		1 (0—3)	1 (0—2)	0.15
Injury Severity Score ISS	total	22 (14—29)	25 (16—30)	0.289
Abbreviated Injury Score	Head and Neck	3 (3—4)	4 (3—5)	0.072
	Facial	0 (0—2)	0 (0—1)	0.783
	Chest	0 (0—3)	0 (0—2)	0.564
	Abdomen	0 (0—0)	0 (0—0)	0.801
	Extremity, pelvis	0 (0—2)	0 (0—2)	0.299
	Other	1 (0—1)	1 (0—1)	0.313
First preclinical GCS		13 (7—15)	13 (6—15)	0.257
Outcome				
Alive at discharge		102 (72.3%)	52 (64.2%)	0.264

^*^Data shown as number (%) or as median (IQR)

**Table 6:** Older population: Trauma setting, mechanism, risk behaviour and co-medication

Trauma setting		Male (63.5%)	Female (36.5%)	*p*-value
Rural		25 (17.7%)	9 (11.1%)	0.261
Urban		30 (21.3%)	15 (18.5%)	0.75
Home environment		55 (39.0%)	47 (58.0%)	**0.009**
Indoor		53 (37.9%)	44 (54.3%)	**0.025**
Trauma mechanism				
Road traffic accident	total	42 (29.8%)	16 (19.8%)	0.216
	car	8 (5.7%)	5 (6.2%)	
	motorcycle	10 (7.1%)	0 (0.0%)	
	e-scooter	1 (0.7%)	0 (0.0%)	
	bicycle	17 (12.1%)	1 (1.2%)	
	e-bike	3 (2.1%)	1 (1.2%)	
	pedestrian	3 (2.1%)	9 (11.1%)	
Railway accident		3 (2.1%)	0 (0.0%)	
Fall	low energy fall < 2 m	70 (49.6%)	52 (64.2%)	0.051
	high energy fall > 2 m	23 (16.3%)	8 (9.9%)	0.258
Delict		1 (0.7%)	0 (0.0%)	
Gunshot		2 (1.4%)	0 (0.0%)	
Burial/Avalanche		0 (0.0%)	1 (1.2%)	
Risk behavior and co-medication				
Under influence of alcohol		20 (14.2%)	5 (6.2%)	0.11
Under influence of other substances		2 (1.4%)	0 (0.0%)	
Self-inflicted injury		4 (2.8%)	3 (3.7%)	0.708
Not belted		1 (12.5%)	1 (20.0%)	0.928
Not helmeted	total	7 (23.3%)	1 (50.0%)	0.711
	motorcyclist	0 (0.0%)	0 (0.0%)	
	e-scooter	0 (0.0%)	0 (0.0%)	
	cyclist	7 (41.2%)	0 (0.0%)	
	e-bike cyclist	0 (0.0%)	1 (100.0%)	
Prior medication	psychotropics	35 (24.8%)	22 (27.2%)	0.823
	anticoagulation	26 (18.4%)	19 (23.5%)	0.47
	antiaggregation	61 (43.3%)	20 (24.7%)	**0.009**

^*^Data shown as number (%)

## Discussion

### Sex-related differences

Our results confirm that sex and age-related differences exist and are in line with previous studies referring to patients with severe TBI in other high income countries (HIC) [2, 4, 33–37]. TBI remains a male predominant patient population across all age groups [2]. With respect to trauma mechanism, we observed distinct sex and age-related differences. Overall, the most common cause of TBI was RTAs in men and low energy falls in women. However, this difference was only observed in the patient population as a whole. Younger patients primarily suffered RTAs and older patients primarily suffered from low energy falls irrespective of sex. The incidence of being under the influence of alcohol when the TBI occurred was higher in men suggesting an increased affinity for risk-taking behaviour. Both alcohol-consumption and stress are associated with higher cortisol levels which in turn has been linked to enhanced risk taking in men but not in women [38–40]. Interestingly, over half of the women compared to less than 25% of men were not helmeted in two-wheeled RTAs.

Women of all ages were more likely to suffer a TBI in the home environment compared to men. The women in this study population were older and more commonly lived independently at home, which could explain this observation. Fall-rate increases with age and remains higher in women with the majority resulting from low energy falls. Furthermore, younger women had a significantly higher rate of previous psychiatric diagnoses and psychiatric co-medication. This may affect trauma incidence, as psychotropic medications are known for increasing the rate of falls [41].

### Changing demographics of TBI in Switzerland

There is a shift in global causality of TBI from RTAs to falls, with a continued male predominance and an increasing sex-heterogeneity with age [10, 35]. Previous epidemiologic studies in Switzerland [15, 16] have mirrored these changes and our data further supports this trend. When comparing our study cohort with the most recent Swiss population-based study by Walder et. al. [15], notable parallels are apparent in terms of median age, sex, and age distribution, as well as the distribution of causative factors, with falls being the most common causator of TBI in our cohort, followed by RTAs.

### Implications for preventive measures

Based on the presented results, sex specific prevention strategies should be developed and their effects studied. Prevention in men will be most effective if risky behaviour, particularly in younger male road users, is targeted. With the increasing use of two-wheeled e-mobility, especially e-bikes and e-scooters, and their typical injury patterns with even higher rates of TBI [42–44], the importance of wearing a helmet cannot be understated [45]. For example, in this study, none of the patients (all were younger males, n = 6) who suffered an e-scooter accident wore a helmet. This mirrors the data seen in other HIC patient populations [46–49]. Without stricter traffic regulations and mandatory helmet laws, TBI incidence in this group is likely to rise [45, 49]. It is of particular note that overall helmet use was particularly low in women in this patient population, with almost 60% of women not having worn a helmet, underscoring the need for targeted road traffic surveillance in this group also.

Women are most likely to suffer from falls within the home environment irrespective of age, while, in males, this mechanism of injury only becomes the most common cause of TBI in older patients. Focus should continue to be placed on fall prevention in the home environment (removal of tripping hazards, use of handrails, housing on one level etc.). Frailty reduction through exercise intervention [50] might prove beneficial. Rigorous reviews and reduction of co-medications which increase fall rate should be considered[41, 51]. Finally, especially in the very old population, the use of antiaggregation or anticoagulation should be critically assessed as they present an ever-increasing risk factor in severe TBI [52].

### Strength and limitations of the study

The patient population was collected using a database of consecutive TBI patients over a 4-year period treated in the trauma ICU of a high-volume trauma centre with standard treatment guidelines of clinical care for TBI through which internal treatment bias was reduced. Extensive data collection and few missing data enabled a high patient inclusion rate. A few study characteristics limit the generalizability of the study, namely the retrospective nature, single centre data collection and the study population originating from a highly developed country with high safety standards. Because our inclusion criteria were limited to patients admitted to the ICU within the first 24 h of hospital admission, cases of secondary or delayed-onset TBI, as well as mild TBI not requiring ICU care, were not captured. Lastly, we abstained from performing a multivariable analysis since our interest was the description of the cohort rather than evaluating whether the differences are relevant for outcome.

## Conclusion

Similar to previously reported HIC patient populations, TBI in Switzerland is male predominant. Sex-related epidemiologic differences in TBI were observed. Men most commonly suffered from a RTA trauma mechanism, while women most commonly suffered from low energy falls. Both sexes exhibit risk-associated behaviour. Men demonstrated a higher incidence of being under alcohol while women were frequently not helmeted. Our results suggest that sex and age-specific prevention have great potential impact to mitigate TBI outcomes.

## Data Availability

No datasets were generated or analysed during the current study.
